# Prevalence and predictors of injuries in Kenya: findings from the national STEPs survey

**DOI:** 10.1186/s12889-018-6061-x

**Published:** 2018-11-07

**Authors:** Gladwell Koku Gathecha, Christine Ngaruiya, Wilfred Mwai, Ann Kendagor, Scholastica Owondo, Loise Nyanjau, Duncan Kibogong, Wilson Odero, Joseph Kibachio

**Affiliations:** 1 Division of Non Communicable Diseases, Ministry of Heath, Nairobi, Kenya; 2 Department of Emergency Medicine, Yale School of Medicine, New Haven, CT USA; 3 Directorate of Road Safety, National Transport and Safety Authority, Nairobi, Kenya; 4 Faculty of Medicine, Maseno University, Kisumu, Kenya; 5 The Institute of Global Health, Faculty of Medicine, University of Geneva (UNIGE), Geneva, Switzerland

**Keywords:** Kenya, Injuries, Logistic regression, Population based survey, Risk factors

## Abstract

**Background:**

Injuries are becoming an increasingly important public health challenge globally, and are responsible for 9% of deaths. Beyond their impact on health and well-being, fatal and non-fatal injuries also affect social and economic development for individuals concerned. Kenya has limited data on the magnitude and factors associated with injuries. This study sought to determine the magnitude and risk factors for injuries in Kenya and to identify where the largest burden lies.

**Methods:**

A national population-based household survey was conducted from April–June 2015 among adults age 18–69 years. A three-stage cluster sample design was used to select clusters, households and eligible individuals based on WHO guidelines**.** We estimated the prevalence of injuries, identified factors associated with injuries and the use of protective devices/practices among road users. Multivariate logistic regression was used to identify potential factors associated with injuries.

**Results:**

A total of 4484 adults were included in the study. Approximately 15% had injuries from the past 12 months, 60.3% were males. Four percent of the respondents had been injured in a road traffic crash, 10.9% had experienced unintentional injuries other than road traffic injuries while 3.7% had been injured in violent incidents. Among drivers and passengers 12.5% reported always using a seatbelt and 8.1% of the drivers reported driving while drunk. The leading causes of injuries other than road traffic crashes were falls (47.6%) and cuts (34.0%). Males (*p* = 0.001), age 18–29 (*p* < 0.05) and smokers (*p* = 0.001) were significantly more likely to be injured in a road traffic crash. A higher social economic status (*p* = 0.001) was protective against other unintentional injuries while students had higher odds for such types of injuries. Heavy episodic drinking (*p* = 0.001) and smoking (*p* < 0.05) were associated with increased likelihood of occurrence of a violent injury.

**Conclusions:**

Our study found that male, heavy episodic drinkers, current smokers and students were associated with various injury types. Our study findings highlight the need to scale up interventions for injury prevention for specific injury mechanisms and target groups. There is need for sustained road safety mass media campaigns and strengthened enforcement on helmet wearing, seatbelt use and drink driving.

## Background

Injuries are becoming an increasingly important public health challenge globally, responsible for 9% of deaths [1]. The mortality from injuries is nearly twice that from HIV, malaria and tuberculosis yet the global efforts have not responded adequately to reduce this burden [1]. The Global Non Communicable Diseases (NCD) Action Plan [2] does not include injuries in its scope unlike the Brazzaville declaration on NCDs and the Kenya National Strategy for NCDs and Injuries [3]. Injuries were intentionally included after realizing the significant burden they produce in the region and the slow progress experienced in reducing this burden despite the availability of proven evidence-based strategies [4, 5]. This is a commendable step towards prioritizing efforts towards injury prevention and control in the African region which bears the largest brunt of deaths due to injuries [1]. It should also be noted that projection for injuries are estimated to be worse for low and middle income countries [6]. More recently, the Sustainable Development Goals have helped to establish priority areas of focus for public health upto the year 2030 [7]. Goal three focuses on NCDs, with a specific target pertaining to injury: to halve the number of deaths and injuries due to road traffic accidents.

Fatalities from injury represent only a small fraction of those who are injured. The Injury pyramid is often used to depict the demand on the health sector caused by injury whereby injury fatalities are at the apex and the base is represented by injuries treated at the community, not treated or not recorded [1]. There are numerous non-fatal injuries which end up in hospital visits and admissions. Additionally, some individuals end up with permanent disabilities and other non-physical consequences including mental illness such as depression, alcohol and drug use dependence and anxiety [1]. In particular, violence such as child maltreatment, intimate violence and collective violence are associated with increased likelihood of mental disorders [1]. The non fatal injuries are important as they result to direct medical costs to hospitals and indirect costs due to loss of productivity [4]. Beyond their impact on health and well being, fatal and non-fatal injuries also affect social and economic development [1, 8].

Kenya has limited data on the magnitude and predictors of injuries. The little available data is limited in geographical scope and no assessments have been carried out on a national scale. Furthermore, while some studies have focused on mortality estimates there has been less emphasis placed on morbidity due to injuries [9–12]. Currently, no population-based survey has been conducted as existing studies have been conducted in a hospital setting. Studies conducted in hospital settings lack representativeness as the severe cases tend to be captured more and it is difficult to ascertain the community population at risk [13].

Available data show that injury is amongst the top ten causes of death in adulthood with Road Traffic Injuries (RTIs) as the leading cause [9–12]. According to data from the National Police Service, approximately 3000 deaths occur annually as a result of RTIs but this estimate is affected by underreporting as deaths occurring at the hospitals are not recorded [14]. A pilot study that was carried out to determine the feasibility of implementing a trauma registry at the main national referral hospital over a period of 12 months found the leading injuries were due to RTI (27.6%), assault (26%) and burns (25.4%) [15].

Risk factors for injuries have been identified in previous studies. As the population increases, migration to urban areas and motorization continue to rise and this has an effect on the rising the number of RTI [16, 17]. Alcohol and drug use have also been associated with increased incidence of injuries [1, 18]. The Literature shows a higher preponderance of injury occurrence among males compared to females [9, 10].

Wearing of helmets and seat belts has been shown to decrease the risk of head injuries experienced during RTIs and to prevent death. A reduction of risk of death of between 42 and 69% related to helmet use has been noted in literature [19]. Similarly the proper use of seat belts decreases the risk of death and severity of injuries among motor vehicle occupants [20, 21]. There are other environmental strategies that can be used such as designated walk ways, zebra crossings and foot bridges to reduce the incidents of road traffic injuries [22].

With evidence of a significant burden due to injury, and a need for nationally representative data the STEPS survey provides information to identify where the largest burden lies. The study additionally provides evidence as a baseline before implementation of the National Violence and Injury Prevention Action Plan 2018–2022 [23]. We performed a detailed analysis of the STEPS 2015 survey data to determine the magnitude and risk factors of injuries in Kenya to obtain information that is crucial in addressing the public health burden of injury in Kenya.

## Methods

STEPwise surveillance for NCD risk factors is a survey methodology developed by WHO to enable countries to collect information that is useful in program planning. The survey contains an optional module on injuries and violence which provided the data that was used in the analysis for this paper. The survey was conducted among adults aged between 18 and 69 years during the period April–June 2015. The STEPwise approach uses a sequential process to collect information. The first step involves conducting interviews to gather information on demographic variables, selected behavioral risk factors and injuries. The second step involves collecting information on anthropometric measurements such as weight, height and blood pressure. The third step involves the collecting of biochemical measurement such as fasting blood glucose and is usually done the following day after step one and two have been done. Data collection was done using a Personal Digital Assistance (PDA) which was loaded with the eSTEPs questionnaire. STEPs was a nationally representative cross-sectional household survey that used the National Sample Survey and Evaluation Programme V (NASSEP V) household sampling frame for sampling determined by the Kenya National Bureau of Statistics. A three-stage cluster sample design was used to select clusters, households and eligible individuals. One individual was selected in the households. Details of its methods have been described elsewhere [24].

An injured person was described as one who had either been injured seriously in a road traffic crash (road traffic injury), had other serious unintentional/accidental injury (accidental injury), or had been seriously injured in a violent incident (violent incident) within the preceding 12 months. Unintentional/accidental injuries included injuries such as a fall, a cut or other injury such as poisoning, near drowning, electrocution. Violent incidents included intentional use of physical force resulting to injury. A serious injury was defined as an injury that required medical attention. This variable included the following questions: “did you have any injuries in this road traffic crash which required medical attention?”, “in the past 12 months, were you accidentally injured, other than the road traffic crashes which required medical attention?”, and “in the past 12 months, how many times were you in a violent incident in which you were injured and required medical attention?”. Binge drinking (heavy episodic drinking) was also defined as drinking six or more standard drinks in a single drinking occasion. Smoking included the use of smoked tobacco products such as cigarettes, hand-rolled cigars, water pipes/shisha, or pipes/*kiko* in the past 30 days. The independent variables used in this study include social demographic variables (sex, age, highest level of education, marital status, occupation), behavioral factors; current smoking and heavy episodic drinking, and physical measurement; body mass index. The outcome variable for this study was reporting of a serious injury in the past 12 months.

All the responses produced in the paper are based on weighted data. Sampling weights were calculated as the inverse or reciprocal of all the selected probabilities. The weights were derived from NASSEP V and the individuals’ selection after adjusting for individual non response. Post stratifications were done to align with the population projections according to age-sex categories.

We assessed the magnitude of the problem of injuries in the country by estimating the prevalence of the occurrence of the three types of injuries mentioned above. We also calculated the prevalence of protective factors for road traffic injuries particularly; seatbelt use, helmet wearing and crossing in designated areas. We conducted descriptive analyses to examine the demographic characteristics of injured respondents and place of occurrence of the injuries. First, we used the *svy* command in Stata software with the over option to get the averages and proportions for various groups of interest. Next, the odds ratio along with its confidence intervals were estimated using generalized linear models for the binomial family which accounts for analytical weights. Adjusted odds ratios were computed for each exposure variable while controlling for all the other variables (confounders) in the model. All socio-economic factors, behavioral factors and physical measurements were included in the final model. *P*-Values of less than 0.05 were considered significant. The STEPs survey had 4500 respondents but for this paper we present data for 4484 respondents after omitting records that had missing values for the independent and dependent variables.

## Results

A total of 4484 individuals participated in the study of which 15.2% reported having been injured in the past 12 months (Table 1). There was an almost equal proportion of women and men who participated in the study; however more men (60.3%) than women (39.4%) reported having been injured (*p* = 0.000). Respondents with no formal education had the lowest proportion of injuries (8.2%). Injuries were experienced more among the respondents residing in rural areas (69.2%) than urban areas (30.9%).

**Table 1:** Socio demographic characteristics of the injured

Category	Injured (n (%))	Not Injured (n (%))	Total number
Sex			
Male	411 (60.3)	1775 (46.7)	2186 (49.8)
Female	269 (39.4)	2029 (53.4)	2298 (51.2)
Age groups			
18–29	287 (42.2)	1775 (46.7)	2062 (46.0)
30–39	162 (23.8)	883 (23.2)	1045 (23.3)
40–49	123 (18.0)	572 (15.0)	695 (15.5)
50–59	78 (11.4)	365 (9.6)	443 (9.9)
60–69	30 (4.4)	209 (5.5)	239 (5.3)
Education level			
No formal education	56 (8.2)	507 (13.3)	563 (12.6)
Primary incomplete	218 (32.0)	826 (21.7)	1044 (23.3)
Primary complete	155 (22.7)	845 (22.2)	1000 (22.2)
Secondary and above	252 (37.0)	1625 (42.7)	1877 (41.9)
Residence			
Rural	472 (69.2)	2304 (60.6)	2776 (61.9)
Urban	207 (30.9)	1501 (39.5)	1708 (38.1)
Occupation			
Unemployed	91 (13.4)	403 (10.6)	494 (10.0)
Employed	414 (61.0)	2289 (60.2)	2703 (60.3)
Student	56 (8.2)	250 (6.6)	306 (6.8)
Homemaker	118 (17.4)	863 (22.7)	981 (21.9)
Marital status			
Not married	155 (22.8)	884 (23.2)	1039 (23.2)
Married	432 (63.6)	2506 (65.9)	2938 (65.5)
Formerly married	93 (13.7)	414 (10.9)	507 (11.3)
Total	679 (15.2)	3805 (84.9)	4484 (100.0)

Nearly 4 % of the respondents had been injured in a road traffic crash as shown in Table 2. The majority of those injured in a road traffic crash were passengers (47.9%). The top three causes of injuries other than road traffic injuries were cuts (47.7%), falls (33.8%) and other injuries (9.6%). Approximately, 3.7% of the respondents reported that they were injured in a violent incident. Men and rural residents had a higher proportion of all categories of injuries than female and urban residents respectively with the exception of burns which were higher among females than men.

**Table 2:** Mechanism of injury

Cause of injury	Percentage				Total number	
	Male	Female	Rural	Urban	N (%)	% (*n* = 4484)
Road traffic						
Driver	100.0	0.0	51.7	48.3	41 (25.2)	
Passenger	59.8	40.3	57.3	42.7	78 (47.9)	
Pedestrian	61.2	38.8	56.9	43.1	11 (6.7)	
A cyclist	94.0	6.0	72.0	28.0	33 (20.2)	
*Total*	*76.9*	*23.1*	*58.9*	*41.1*	163 (100.0)	*3.6*
Accidental injury						
Cut	59.7	40.3	73.5	26.5	234 (47.7)	
Fall	54.0	46.0	70.4	29.6	166 (33.8)	
Others	60.6	39.4	61.3	38.7	47 (9.6)	
Animal bite	59.0	41.0	84.9	15.1	25 (5.1)	
Burn	33.0	67.0	88.9	11.1	19 (3.9)	
*Total*	*56.8*	*43.2*	*72.4*	*27.6*	*491* (100.0)	*10.9*
Violent incident						
With weapon	56.7	43.3	70.9	29.1	59 (35.5)	
No weapon	71.5	28.5	66.2	33.8	86 (51.8)	
Others	58.4	41.6	78.8	21.2	21 (12.7)	
*Total*	*64.6*	*35.4*	*69.3*	*30.7*	*166* (100.0)	*3.7*

Table 3 highlights the place of injury occurrence. Two fifths (40.7%) of the injuries occurred at home followed by farm (16.9%) and work place (16.7%). The commonly occurring type of injury in schools is falls. The majority of the burn injuries occurred at the work place (5.5%).

**Table 3:** Place of occurrence of Injury for other unintentional injuries

Place	Fall %	Burn %	Cut %	Animal bite %	Other %	Total number (%)
Home	31.4	5.2	49.3	9.7	4.4	200 (40.7)
Farm	7.8	0.0	88.4	1.9	2.0	83 (16.9)
Workplace	26.5	5.5	46.2	3.2	18.7	82 (16.7)
Road	56.7	4.5	20.6	1.4	16.8	69 (14.1)
Others	45.6	4.0	28.5	0.0	21.9	26 (5.3)
School	90.1	0.0	8.1	0.0	1.9	22 (4.5)
Sports	56.6	0.0	0.0	0.0	43.4	9 (1.8)
Total	33.9	3.9	47.6	5.0	9.6	491 (100.0)

The study found the proportion of protective behavior among road users to be low as shown in Table 4. Nearly half (49.1%) of all respondents said they never use seat belts and 62% of those who use motorcycles do not use helmets. Approximately, 8.1% of all drivers admitted to driving while under the influence of alcohol. Only 15.8% of the respondents said they always used designated crossing areas to cross the road.

**Table 4:** Prevalence of protective and risk factors for road safety

Protective factor	Road Traffic Accident		
	No (n (%))	Yes (n (%))	Total (n (%))
Seat belt use			
All of the time	405 (12.5)	15 (14.0)	420 (12.5)
Sometimes	1016 (31.2)	29 (28.1)	1046 (31.1)
Never	1596 (49.1)	51 (49.2)	1647 (49.1)
Seat belt not present	236 (7.3)	9 (8.7)	245 (7.3)
*Total*	*3253 (96.9)*	*105 (3.1)*	*3358*
Helmet wearing			
All of the time	186 (5.5)	21 (16.3)	206 (5.9)
Sometimes	316 (9.4)	20 (16.0)	336 (9.6)
Never	2101 (62.6)	60 (47.1)	2160 (62.0)
Helmet not present	756 (22.5)	26 (20.6)	782 (22.4)
*Total*	*3358 (96.4)*	*127 (3.6)*	*3485*
Ride on motorbike while drunk			
Always	128 (4.4)	20 (20.2)	148 (4.9)
Sometimes	205 (7.1)	9 (9.5)	215 (7.1)
Never	2583 (88.6)	69 (70.2)	2652 (88.0)
*Total*	*2916 (96.7)*	*98 (3.3)*	*3014*
Drove vehicle while drunk			
No	3977 (91.7)	134 (96.4)	4111 (91.9)
Yes	359 (8.3)	5 (3.6)	364 (8.1)
*Total*	*4336 (96.9)*	*139 (3.1)*	*4475*
Designated road crossing areas			
Every time	626 (15.9)	17 (13.2)	643 (15.8)
Sometimes	741 (18.8)	29 (22.1)	770 (18.9)
Never	913 (23.2)	41 (31.2)	954 (23.5)
No designated area	1655 (42.1)	44 (33.5)	1699 (41.8)
Total	3935 (96.8)	130 (3.2)	4065

Factors associated with injuries are highlighted in Table 5. Road traffic injuries were associated with being male (OR = 3.87; 95% CI = 2.24–6.68), being of younger age: 18–29 age group (OR = 2.96; 95% CI = 1.48–5.90), and 40–49 age group (OR = 2.77; 95% CI = 1.38–5.55) having the highest odds. Current smokers were also more likely to have been involved in a RTI (OR = 2.08; 95% CI = 1.31–3.32). Furthermore, students (OR = 2.00; 95% CI = 1.24–3.22) and individuals who were underweight (OR = 1.57; 95% CI = 1.18–2.09) were more likely to be involved in other accidental injuries. Being in the richest quintile (OR = 0.32; 95% CI = 0.20–0.50) and the fourth quintile (OR = 0.43; 95% CI = 0.29–0.63) were protective against encountering other accidental injuries. Violent incidents were experienced more among respondents age 30–39 years (OR = 1.76; 95% CI = 1.04–2.99), binge drinkers (OR = 2.34; 95% CI = 1.57–3.49) and current smokers (OR = 1.73; 95% CI = 1.14–2.63). Respondents who were obese were less likely to be involved in a violent incident (OR = 1.73; 95% CI = 1.14–2.63).

**Table 5:** Factors associated with injuries

Predictor	Road traffic injuries		Accidental injury		Violent incident	
	OR (95% CI)	P > z	OR (95% CI)	P > z	OR (95% CI)	P > z
Sex						
Female	1.00		1.00		1.00	
Male	3.87 (2.24, 6.68)	0.000	1.24 (0.96, 1.61)	0.096	1.17 (0.76, 1.79)	0.485
Residence						
Rural	1.00		1.00		1.00	
Urban	1.20 (0.77, 1.86)	0.425	0.89 (0.67, 1.17)	0.392	1.14 (0.75, 1.74)	0.551
Age group						
50–69	1.00		1.00		1.00	
18–29	2.96 (1.48, 5.90)	0.002	0.82 (0.59, 1.13)	0.228	1.03 (0.59, 1.81)	0.919
30–39	1.65 (0.80, 3.41)	0.174	0.89 (0.65, 1.23)	0.482	1.76 (1.04, 2.99)	0.035
40–49	2.77 (1.38, 5.55)	0.004	0.96 (0.68, 1.35)	0.806	1.32 (0.73, 2.37)	0.361
BMI						
Normal	1.00		1.00		1.00	
Underweight	0.82 (0.45, 1.52)	0.536	1.57 (1.18, 2.09)	0.002	1.45 (0.94, 2.23)	0.090
Overweight	0.93 (0.55, 1.60)	0.804	1.18 (0.89, 1.56)	0.260	0.78 (0.47, 1.31)	0.354
Obese	1.78 (0.95, 3.34)	0.073	1.03 (0.68, 1.55)	0.905	0.28 (0.09, 0.89)	0.031
Episodic alcohol drinking						
No alcohol	1.00		1.00		1.00	
Binge	1.15 (0.73, 1.80)	0.547	1.27 (0.95, 1.70)	0.105	2.34 (1.57, 3.49)	0.000
Non-heavy	0.55 (0.22, 1.39)	0.208	0.70 (0.42, 1.17)	0.172	0.88 (0.41, 1.88)	0.736
Education level						
No formal education	1.00		1.00		1.00	
Primary education	2.29 (0.83, 6.32)	0.110	2.52 (1.69, 3.77)	0.000	1.13 (0.65, 1.97)	0.662
Secondary and above	1.78 (0.62, 5.10)	0.285	2.57 (1.65, 4.01)	0.000	0.53 (0.27, 1.03)	0.063
Occupation						
Unemployed	1.00		1.00		1.00	
Employed	1.18 (0.64, 2.18)	0.597	1.08 (0.78, 1.49)	0.641	1.05 (0.65, 1.70)	0.833
Student	0.63 (0.19, 2.12)	0.456	2.00 (1.24, 3.22)	0.005	1.51 (0.67, 3.43)	0.319
Homemaker	1.52 (0.68, 3.38)	0.306	0.92 (0.62, 1.36)	0.671	0.70 (0.37, 1.32)	0.273
Wealth Quintile						
Poorest	1.00		1.00		1.00	
Second	0.88 (0.41, 1.91)	0.753	0.68 (0.5, 0.92)	0.014	1.37 (0.84, 2.22)	0.205
Middle	2.76 (1.40, 5.45)	0.003	0.82 (0.60, 1.13)	0.228	1.01 (0.58, 1.74)	0.977
Fourth	1.68 (0.80, 3.53)	0.170	0.43 (0.29, 0.63)	0.000	1.00 (0.55, 1.83)	0.999
Richest	1.04 (0.44, 2.44)	0.930	0.32 (0.20, 0.50)	0.000	0.55 (0.26, 1.15)	0.114
Marital status						
Not married	1.00		1.00		1.00	
Married	2.09 (1.18, 3.69)	0.011	1.13 (0.82, 1.55)	0.459	0.8 (0.51, 1.27)	0.350
Formerly married	2.68 (1.29, 5.57)	0.008	1.62 (1.08, 2.44)	0.020	0.93 (0.51, 1.69)	0.811
Smoke						
No	1.00		1.00		1.00	
Yes	2.08 (1.31, 3.32)	0.002	1.17 (0.84, 1.61)	0.348	1.73 (1.14, 2.63)	0.011

## Discussion

This population-based survey provides the first national estimates on non-fatal injuries in Kenya. Our study additionally identified factors associated with injuries that include social, demographic, behavioral and biological. This is important as it helps to identify targeted interventions that could have a greater impact in reducing the burden of injuries. Fifteen percent of the respondents reported that they had been seriously injured in the past 12 months and required medical attention. This proportion is quite high and needs to be addressed as injuries have far reaching effects on individuals, societies and the health care systems [1, 8]. This is higher than what has been reported in developed countries such as Germany with 10.7% [25]. and developing countries such as Sierra Leone with 12.4% [26] and Uganda 14% [27].

Our findings are consistent with other studies that have found a higher risk of injuries among males than females [26, 28, 29]. The gender disparity in injury mortality, has been attributed to multiple factors and the same concepts can be advanced for the gender disparities in non fatal injuries [30]. There is increased exposure to injury risk factors such as alcohol use and occupational hazards among males compared to females [24].

Research findings also reveal the great role societal factors play in injury incident such as level of masculinity and perceived gender roles which in turn have a bearing on risk taking [31]. Masculinity is described as a set of characteristics, qualities or roles that are generally attributed to men [32]. The high burden of injury in males needs to be addressed. In addition we must also recognize that though prevalence of injury among females is low, injuries are still an important cause of morbidity and mortality in this group [10]. In addition to public health interventions that advocate for behaviour change such as stoppage of alcohol drinking, measures that advocate for societal changes must also be heightened.

The prevalence of injuries was significantly higher among rural residents than urban residents. Previous studies corroborate this finding [33, 34]. Other studies have found no difference in occurrence of injury by place of residence [26, 28]. Some studies conducted among children have found a higher preponderance of injuries among children living in urban areas [35]. The occurrence of injury in rural settings may be explained by the nature of work related activities that include farming which could carry a higher risk of injury. Majority of the country’s population reside in rural areas with faming as their primary mainstay, this difference in incidence of injuries between the two areas has to be factored in when setting priorities for injury prevention interventions in the country.

Injuries have been widely associated with low social economic status [5, 28]. A report released by the World Bank in January 2018 estimated that countries can lose between 7 and 22% in potential per capita gross domestic growth over a 24-year period [36]. In our study, this pattern was true for only other accidental injuries where respondents from the two richest quintiles had less likelihood of getting other unintentional injuries. Individuals from poorer backgrounds tend to live, work and travel in less safe conditions [1].

The strongest association between injuries and heavy episodic drinking (binge) was found among respondents who had been involved in a violent incident. Similar findings have been found elsewhere [18, 37, 38]. It should however be noted that our study did not directly link the injury to alcohol taking prior to the injury. However, frequency of alcohol taking is a good proxy to estimate alcohol related injury. Our study seemingly contradicted the preventive paradox theory which states that more alcohol related harm is found among the drinkers at low risk [39]. Respondents who drank alcohol but were not binge drinkers tended to have fewer injuries than binge drinkers and non-alcohol drinkers though this was not statistically significant. Nevertheless, alcohol control policies including drink driving measures need to be stepped up in the country. In particular domestication of the global strategy for harmful reduction of alcohol use needs to be fast tracked [40].

Our study explored the association between injury and smoking, an area with limited information. Respondents who smoke were more likely to be involved in a road traffic injury and violent injury than respondents who did not smoke. Prior studies have found evidence of this relationship between smoking and risk of injuries [41, 42]. It has been advanced that the association between smoking and injury is as a result of direct toxicity, distractibility, smoking-associated medical conditions and confounding factors, including personality or behavioral characteristics [41]. There is need to heighten public health awareness to healthcare workers and the public on this additional harmful association of smoking.

Road traffic injuries have been a long standing public health concern in Kenya [14, 43]. Nearly 4 % of the respondents had been involved in a road traffic crash and sustained injuries that required medical attention. Surprisingly there were a higher proportion of respondents injured in the rural areas than urban areas where the level of motorization is higher. It is possible that the a high influx of motorcycles and bicycles in the rural areas could be responsible for this [44]. The consistent use of seat belt and helmets was reported by 13% and 6% of the respondents respectively. A study conducted in two towns in Kenya, Thika and Naivasha, found the prevalence of helmet use among riders to be 25.7% and 37.2% respectively [45]. These levels remain worryingly low despite the numerous heightened mass media campaigns conducted by various road safety agencies. To bring meaningful change in this area, enforcement efforts have to be strengthened alongside the media campaigns. There was an admission of driving while drunk and riding while drunk by 8% and 7% of the drivers and riders respectively. The Traffic Amendment Act Cap 405 prohibits drink driving. There needs to be strengthened enforcement on drink driving and also the law needs to be amended to prohibit riders from riding under the influence of alcohol.

Nearly one in ten of the respondents had been involved in an unintentional injury other than road traffic injury. Cuts were the most common injury type followed by falls. Other studies have found falls to be the most frequently occurring injury [26, 29]. The difference in ranking in our study may be attributed to the occupation of the majority of the respondents as most of the cuts occurred at the farm. Nevertheless, these types of injuries are preventable and people should be educated on how to make home, school and work environments safe.

Our study provides the first national estimates for violence among adults in the country other than domestic and sexual violence. Four percent of the respondents reported that they were involved in a violent incident in the preceding 12 months. This is lower than what has been reported in other African counties. Sudan has recorded a prevalence of violence of 7.1% [28] while in Côte d’Ivoire the prevalence ranges between 12.2 and 21% [46]. It is important to note that however the two countries have been afflicted by human conflict in the past. Half of the violence was perpetrated without using a weapon implying that most of the incidents may not have been premeditated. Violence is preventable using the public health approach and therefore the relevant stakeholders need to work together to institute measures [1].

Our study had several limitations. The use of self reporting may have led to underreporting due to recall bias leading to underestimation of injuries. The period used to assess injuries was one year and it is possible that respondents may not have remembered the injuries they sustained. Nevertheless, it has been established that community based studies are less prone to underestimation compared to hospital based studies [13]. Secondly, the study is unable to tell the direction of some associations such as binge drinking. There is also a likelihood of under reporting of some of the variables such as binge drinking and smoking because of the way they are viewed by the society. Underreporting of intentional injuries because of social undesirability may also have occurred. Additionally, the findings of this study underestimate the injury burden because of exclusion of patient outcomes such as disability adjusted life years, years lived with disability and mortality [47]. Furthermore, the study did not assess the societal or economic effects of injuries. Lastly, the study excluded sexually based violence. Despite these limitations, the study gives important insights on the burden of non-fatal injuries in the country. Given the current situation where there is a paucity of data, this is a first step towards gathering information and further studies should be conducted to give comprehensive national information on patient outcomes and socioeconomic effects. The main strength of this study is that it is nationally representative and allows for the exploration of a diversity of associations which are useful for policy formulation.

## Conclusions

The study shows that injuries are an important public health problem in Kenya. This is the first national study in Kenya that explores the risk factors for injuries thereby providing valuable information for targeted prevention initiatives. The information from this study speaks to both policy makers and the public on the significant injury burden in the country. Priority accorded to injury prevention and control remains low with weak national institutional frameworks as in most developing countries that are experiencing a triple burden of disease [48]. Our data supports the need for multiple interventions including actions outside the health sector that are needed to reduce the injury burden in the country. Continued monitoring and enhanced research on injuries are key strategies that should be employed in addressing the injury problem in the country.

## Abbreviations

CDC: Centers for Disease Prevention and Control
HIV: Human Immunodeficiency Virus
KEMRI: Kenya Medical Research Institute
NCD: Non Communicable Diseases
NTSA: National Transport and Safety Authority
OR: Odds Ratio
PDA: Personal Digital Assistant
RTI: Road Traffic Injuries
WHO: World Health Organisation
